# Untangling Sampling Bias From Lemur Dietary Specialization

**DOI:** 10.1002/ece3.72765

**Published:** 2026-01-04

**Authors:** Anna Vasenina, Camille M. M. DeSisto, Peter J. Mucha

**Affiliations:** ^1^ Department of Mathematics Dartmouth College Hanover New Hampshire USA; ^2^ Nicholas School of the Environment Duke University Durham North Carolina USA; ^3^ Rice Sustainability Institute Rice University Houston Texas USA

**Keywords:** functional traits, information completeness, Madagascar, sampling bias, trophic interactions

## Abstract

Identifying the drivers of wildlife dietary specialization is fundamental to understanding trophic interactions and species' functional roles in their environment. However, sampling bias is pervasive in trophic interaction research, especially in biodiverse areas with cryptic species. We aim to investigate the role of sampling bias in mediating the estimated effects of functional traits on dietary specialization. Specifically, we improve estimates of observed lemur dietary richness by analyzing trait‐based biases in lemur–plant ecological interactions. First, we quantified undersampling of plants and their interactions with lemurs. Next, we tested the inclusion of sampling effort on the estimated effects of trait predictors by comparing three negative binomial regression models with the following predictors: (i) five lemur traits (body mass, litter size, group size, diurnality, frugivory), (ii) sampling effort, and (iii) the five traits together with sampling effort. We then assessed the influence of sampling bias on lemur traits and considered the uncertainty of these mediation effects on general linear model predictions. Sampling effort was a better predictor of observed dietary richness than lemur traits. By accounting for sampling bias and its effects on functional traits, we can better understand the ecological roles of species in their environments and uncover critical mechanisms underlying trophic interactions.

## Graphical abstract

Sampling bias may mediate the estimated effects of functional traits on lemur dietary richness. Considering sampling bias and its effects on functional traits is critical for understanding the ecological roles of species in their environments and the mechanisms underlying trophic interactions.

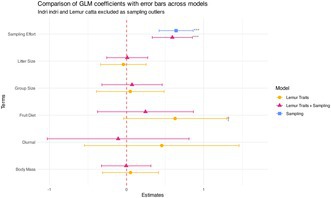

## 1 Introduction

Dietary diversity underpins ecological and evolutionary processes. The number and composition of food species for a given animal influence behavior (Tinker et al. 2008), evolution (Guimarães et al. 2017), and extinction risk (Machado, Jardim, et al. 2023; Reed and Tosh 2019). Conserving trophic interactions is therefore critical for sustaining global biodiversity (Kaiser‐Bunbury and Blüthgen 2015; Heinen et al. 2020). However, one of the main shortfalls that limit large‐scale biodiversity science is the lack of knowledge on biotic interactions (Hortal et al. 2015). Trophic interactions remain poorly understood, and dietary specialization of animals—especially of cryptic species—is poorly characterized (Pringle and Hutchinson 2020). For example, the collection of ecological interaction data is highly concentrated in North America and Europe (Poisot et al. 2021), and therefore systematically neglects global hotspots of biodiversity and extinction (Mittermeier et al. 2011).

The undersampling of certain species has long been considered a fundamental problem in ecological research (Gotelli and Colwell 2001). Taxonomic and geographic biases are pervasive in animal biodiversity studies, and the tropics are particularly poorly sampled (Hughes et al. 2021). Sampling of ecological interactions presents similar challenges to sampling of species; even in studies with high sampling effort, interaction incompleteness persists (Chacoff et al. 2012). Literature compilations of dietary data (e.g., Fricke and Svenning 2020; Steffens et al. 2020) can partially overcome undersampling by combining different data sources in situations where logistics and finances preclude systematic sampling. Incomplete sampling, however, remains an important challenge even in literature compilations (e.g., Tonos et al. (2024)). Although predicting dietary richness through rarefaction and extrapolation methods is conceptually appealing, the inherent uncertainty and sampling bias in current dietary data may undermine the statistical robustness of such analyses. Therefore, understanding underlying bias in dietary data, especially in biodiverse areas with cryptic species, is a priority for ecological research (Papadogeorgou et al. 2023; Kampe et al. 2025).

Functional traits drive both dietary richness and sampling bias. In trophic interaction networks, traits of consumers and their prey act together to influence network structure (Dehling et al. 2016; Bender et al. 2018; Schleuning et al. 2011). For example, large‐seeded plants are more connected to frugivores than small‐seeded plants in southwest China (Li et al. 2020). Large‐bodied animals tend to eat plants with larger fruits, a process called “size matching” (Donatti et al. 2011; Naniwadekar et al. 2019). However, other research has found that, while fruit size is phylogenetically conserved, it was not related to primate food selection, highlighting the importance of evolutionary mechanisms in dietary diversity (Mopán‐Chilito et al. 2022). Size mismatching, as well as spatial and temporal mismatches, can cause “forbidden links” in ecological networks, where species cannot interact. Life history information is therefore critical for assessing the completeness of sampling in dietary data (Jordano 2016). Functional traits also influence patterns of bias in ecological research. For example, among primates, large species are better studied than small species (Machado, Silva Rocha, et al. 2023). To achieve a mechanistic understanding of dietary specialization, it is essential to separate the influence of functional traits on dietary richness itself from their impact on sampling.

Characterizing bias in dietary data and improving estimates of dietary specialization is particularly important in biodiverse areas with high levels of both cryptic interactions and conservation threats, such as Madagascar (Antonelli et al. 2022; Ralimanana et al. 2022). Anthropogenic pressures such as deforestation threaten 94% of extant lemurs (Schwitzer et al. 2014) and 63% of endemic trees (Beech et al. 2021) with extinction. Representing 111 species across five families, the lemur clade is ecologically and functionally diverse. Approximately half of lemur species are known frugivores (Razafindratsima et al. 2019). As Madagascar's primary seed dispersers (Razafindratsima 2014; Albert‐Daviaud et al. 2018), frugivorous lemurs play a critical role in plant community dynamics and biodiversity maintenance. Many lemurs also interact antagonistically with plants by predating upon seeds and eating leaves, bark, etc. (Steffens 2020). Approximately 28% of lemurs, particularly small nocturnal species, are known to consume invertebrates (Razafindratsima et al. 2019). Additionally, the majority (~60%) of lemur species are nocturnal (Razafindratsima et al. 2019).

Lemur functional traits are a critical component of lemur–plant interactions, from ecological network connectivity to seed dispersal outcomes such as germination (DeSisto and Herrera 2022; DeSisto, Zandry, et al. 2025). Lemur traits relating to foraging behavior and energetic requirements likely affect dietary specialization. For example, compared to smaller primates, large primates need more energy, have a greater ability to access food resources, and consume higher quantities of lower quality food (Sailer et al. 1985; Temerin et al. 2019). Larger group size is associated with mammal foraging efficiency (Silk 2007). While the activity pattern may affect lemur foraging ability, the majority of lemur dietary research has been conducted on diurnal species (Steffens 2020). Because specialist species tend to be less tolerant to environmental change (Clavel et al. 2011), conservation status may also help explain lemur dietary specialization. Despite Madagascar's rich history of lemur‐plant interaction research (Steffens 2020; Razafindratsima et al. 2022), knowledge gaps of Madagascar's unique biodiversity persist (Antonelli et al. 2022). In particular, recent research has highlighted that lemur‐plant interaction sampling is heavily skewed to certain sites and species (Tonos et al. 2024). We seek to build on this research, untangling the effects of sampling bias and functional traits on dietary richness to predict lemur dietary specialization.

We analyze sampling‐biased ecological interaction data of lemurs and their food plants to (i) estimate sampling completeness of dietary data with rarefaction and extrapolation and (ii) assess trait‐based biases in sampling and compute the impact of these biases on general linear model predictions of dietary richness. We expect that, in addition to skewed undersampling, trait‐based taxonomic biases influence our estimation of species dietary richness. Several key functional traits, especially lemur body size and activity pattern, are expected to drive both bias in dietary data collection and the biological interactions themselves (Tonos et al. 2024). Robustly characterizing trait‐based biases in dietary data and separating true trait prediction signals from sampling bias effects is valuable for advancing our understanding of natural history and informing sampling methodologies.

## 2 Methods

### 2.1 Data

#### 2.1.1 Lemur Dietary Data

We used lemur dietary data from the Lemur Food Plant database (LFP; Steffens 2020). The LFP is a database of 5420 observed interactions among 56 lemur species and 599 plant genera they consume from 204 individual studies. In the LFP, 58% of plant records were identified to species and 42% to genus. Therefore, following DeSisto and Herrera (2022), we analyzed plants at the genus level to address the low taxonomic resolution of plants in the dataset. This resulted in 55 lemur species and 590 plant genera in the final dataset. Aggregating plant records to the genus level standardizes taxonomic resolution across records, reducing bias in our analysis of how sampling effort influences estimated plant richness. However, aggregating to the genus level may obscure ecological nuances that would be detectable if more plants were identified to the species level.

#### 2.1.2 Traits

We compiled a lemur trait assemblage that represented morphological, life history, and ecological traits which may predict lemur dietary richness. We collected the following lemur traits from Razafindratsima et al. (2018), Razafindratsima et al. (2019), and IUCN (2024): body mass, brain mass, activity pattern (nocturnal or diurnal/cathemeral), diet (frugivore or other), gestation length, group size, habitat (wet, dry, or both), home range size, interbirth interval, and litter size. These life history traits likely affect lemur dietary specialization through energetic needs, foraging behavior, and sociality (DeSisto and Herrera 2022; MacLean et al. 2009; Godfrey et al. 2004). We grouped diurnal and cathemeral species because both have active periods during the day, likely leading to less undersampling of their interactions in the literature. A species was considered a frugivore if there is evidence that it has fruit in its diet (Razafindratsima et al. 2018). We also included conservation threat status according to IUCN categories because specialist species tend to be less tolerant to environmental change (Clavel et al. 2011). Conservation threat status represents composite factors—such as habitat specialization, geographic range size, or human pressures—that are not fully captured by the other measured traits. Species were categorized as endangered (IUCN status of Endangered or Critically Endangered) or not endangered (all other categories) for analysis. Our analysis is correlative and does not infer that these traits directly cause dietary specialization.

Across the 55 lemur species, 90% of these 11 trait values were available in this literature. We imputed the remaining 10% of trait values to obtain a complete trait assemblage using the following approach. Accounting for both trait matrix correlations and phylogenetic relationships, we imputed missing lemur trait values using the *funspace* package (Pavanetto and Puglielli 2023). We constructed the phylogenetic tree of the 55 lemur species used in trait imputations using the *U.PhyloMaker* package (Jin and Qian 2023) and Upham et al.'s (2019) mammal megatree (Upham et al. 2019). All analysis was conducted in R Version 4.3.1 (R Core Team 2023).

Taxonomic sampling effort—or sampling effort per lemur species—was defined as the number of observations recorded in the LFP for any given species (i.e., the number of studies in the LFP which recorded a given species). While this metric is limited in that it does not capture factors such as individual variation in activity patterns or sampling methodology, it standardizes across heterogeneous studies and captures variation in research attention.

### 2.2 Data Processing and Analyses

#### 2.2.1 Sampling Completeness

We characterized sampling completeness of plant genera that lemurs consume and lemur–plant interactions using rarefaction and extrapolation with Hill numbers (Chao et al. 2014). We used Hill‐number‐based rarefaction and extrapolation rather than bipartite network indices because Hill numbers provide a standardized framework that accounts for uneven sampling effort. We computed richness, the exponential of Shannon's diversity, and the inverse of Simpson's diversity, as well as their associated 95% confidence intervals, using sample‐size‐based rarefaction and extrapolation curves. Richness represents the number of unique observations (plant genera and dietary interactions). Shannon's diversity is an index that accounts for both richness and evenness (how evenly individuals are distributed). Simpson's diversity is an index that accounts for both richness and dominance, such that it is less sensitive to rare observations compared to Shannon's diversity. We used the *iNEXT* package (Hsieh et al. 2016) to evaluate plant genera diversity and the *iNEXT.link* package (Chiu et al. 2023) to evaluate interaction diversity. For plant genera, we calculated sampling completeness based on the number of observations, number of studies, and number of sites. For lemur‐plant interactions, we calculated sampling completeness based on the number of observations because *iNEXT.link* is compatible with binary interaction matrices and not incidence matrices.

#### 2.2.2 Trait Dimension Reduction

**FIGURE 1:**
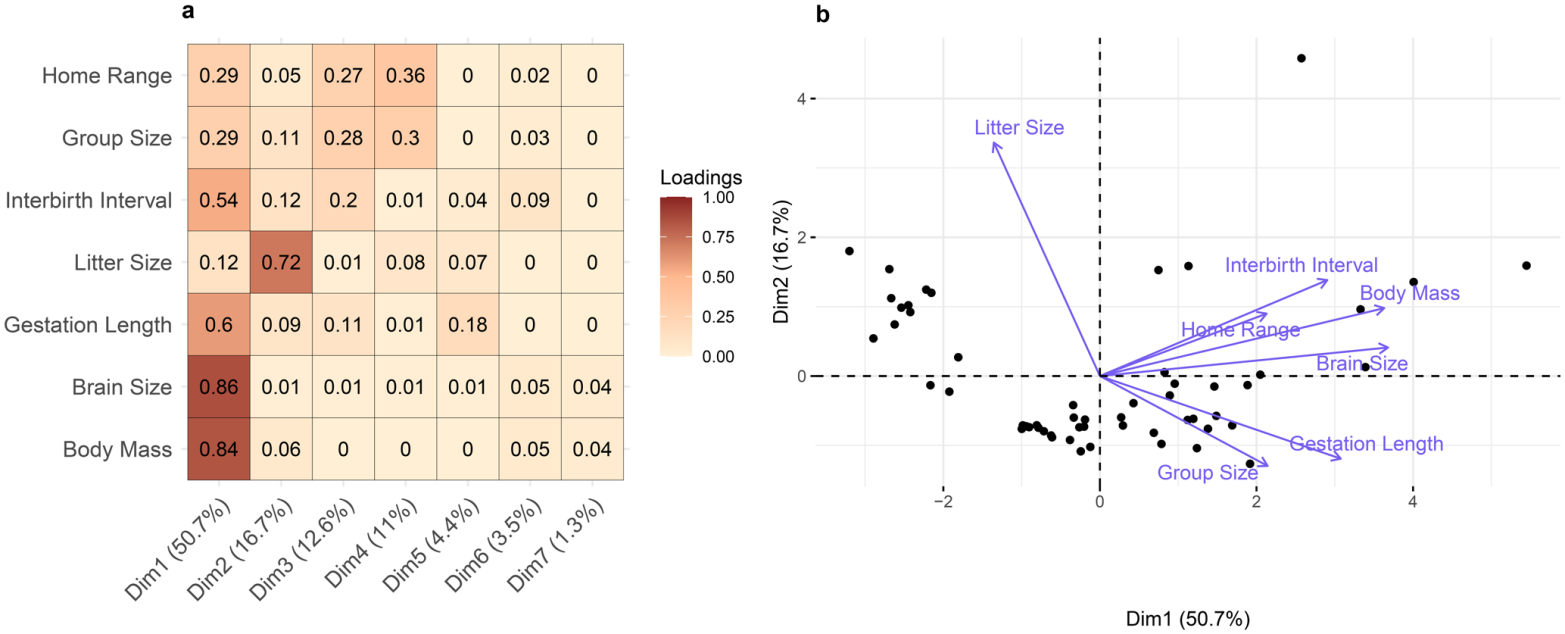
(a) PCA loadings of seven continuous lemur traits: Body mass, brain size, gestation length, litter size, interbirth interval, group size, and home range. Body mass and brain size dominated the first principal dimension of trait variation, while the second mode of trait variation was closely aligned with litter size. Group size appeared equally across the first, third, and fourth modes. (b) PCA biplot of the seven traits: The first two principal components explained 67.4% of trait variation across the 55 lemur species.

We used principal component analysis (PCA) for continuous lemur traits to reduce the number of correlates in our prediction models and identify traits that explain the most variation across all lemur species (Figure 1).

Body mass, group size, and litter size captured the main modes of variation between the first four principal components and were therefore chosen as continuous predictors. Both body mass and brain size were strongly loaded onto the first principal component, and they were highly correlated (correlation = 0.89, *p* < 0.001). We therefore chose to include body mass over brain mass because body mass directly affects energetic requirements, digestive morphology (e.g., gut retention time), and movement (Gaulin 1979; Temerin et al. 2019). Group size and home range size also loaded similarly onto the principal components; we chose to include group size over home range size because primate sociality is associated with improved foraging efficiency, inter‐specific competitive ability, and cognition (Silk 2007; MacLean et al. 2013; Pride 2005; Pride et al. 2006). Although other choices are possible, we believe that they will yield similar results due to similar loadings across the principal components.

Frugivory and diurnality were the only two binary predictors that showed individual clustering in PCA coordinates (see Figure S1), i.e., there was some clustering in lemurs sharing activity patterns and dietary behavior which can be exploited for analysis. In addition, we believe that these traits are prone to taxonomic sampling bias due to ease of observation during the day of diurnal species and research interest in seed dispersal via fruit consumption (Tonos et al. 2024).

#### 2.2.3 Dispersion of Dietary Data

While Poisson models are commonly used and qualitatively appropriate for ecological data, the overdispersion assumption must be checked, i.e., variance should not significantly exceed the mean in our data. We assessed dispersion in the count response of dietary richness by fitting an intercept‐only Poisson model (on *n* = 53 lemurs, excluding outliers, see Section 2.2.4) and computing both the Pearson $\chi^{2}$ dispersion statistic and the deviance divided by residual degrees of freedom. Specifically, Pearson $\chi^{2}$ was calculated as $\sum r_{i}^{2}$ using Pearson residuals $r_{i}$ from the fitted Poisson model, with dispersion defined as Pearson $\chi^{2}/\left(n-1\right)$; the deviance‐based dispersion was computed as model deviance over $\left(n-1\right)$.

For our data, both Pearson dispersion and deviance‐based dispersion were 37, indicating extreme overdispersion relative to the Poisson assumption. We therefore used a negative binomial model that accommodates extra variation instead. See Figures S5–S8 and Tables S9–S14 for results using a Poisson distribution.

#### 2.2.4 Dietary Richness Negative Binomial Model

We removed *Indri indri* and *Lemur catta* species from the model due to known sampling biases (Tonos et al. 2024) (see Tables S5–S8 for results including *L. catta* and *I. indri*, demonstrating that this choice did not strongly impact our conclusions). Both *I. indri* and *L. catta* are conspicuous flagship species that have been the focus of numerous ecological and behavioral studies.

The dependent variable for all our models was observed lemur dietary richness, i.e., the number of plants eaten by a lemur species. Since it is represented in our data by an overdispersed, right‐skewed, heavily‐tailed distribution, we chose a negative binomial general linear model framework for prediction. For all models, Akaike Information Criterion (AIC) and pseudo‐*R* ^2^ (also known as McFadden's *R* ^2^, which was used because it is not possible to compute R‐squared for negative binomial regression models) were used to describe the goodness‐of‐fit across runs. McFadden's *R* ^2^ is defined as $1-\ln\left(L_{M}\right)/\ln\left(L_{0}\right),$ where $L_{M}$ and $L_{0}$ are the likelihoods for the model being fitted and the null model, respectively. It quantifies the proportional improvement in log‐likelihood of the fitted model versus a null model; it is typically much smaller than ordinary *R* ^2^ from linear regression and should be interpreted as a comparative measure of explanatory power (values closer to 0 indicate little improvement over the null, and values closer to 1 indicate greater improvement), rather than as the fraction of variance explained. For all three models, we performed a nonparametric case bootstrap by repeatedly resampling 1000 times (with replacement) 53 species observations from the original dataset and refitting each model to these bootstrapped samples to obtain empirical distributions of AIC and pseudo‐*R* ^2^. For robustness and interpretability of model comparison, we computed the Δ AIC and Akaike weights (wAIC) on the original non‐bootstrapped data for all three negative binomial models (Table 1).

**TABLE 1:** Model selection and relative support: Comparison of lemur dietary richness models. ΔAIC and Akaike weights for a model set consisting of three non‐bootstrapped, negative binomial regression models with differing predictor sets, initialized with a fixed dispersion theta value of 1.205 (see Section 2.2.3).

Model	AIC	Δ AIC	wAIC
Sampling	487.3	0.000	9.887e‐01
Lemur Traits + Sampling	496.2	8.939	1.133e‐02
Lemur traits	508.9	21.614	2.003e‐05

We initialized the negative‐binomial dispersion parameter (Θ) using the method‐of‐moments estimate $\Theta =\mu^{2}/\left(\mathrm{Var}-\mu \right)$, which for our dietary richness data (mean μ = 43.79, Var = 1635.63) gave Θ= 1.205. Θ parametrizes the negative binomial variance via $\mathrm{Var}\left(Y\right)=$ μ +$\frac{\mu^{2}}{\Theta },$ so this value indicates substantial extra‐Poisson variance and was used as a starting value to aid model convergence.

## 3 Results

### 3.1 Sampling Completeness

**FIGURE 2:**
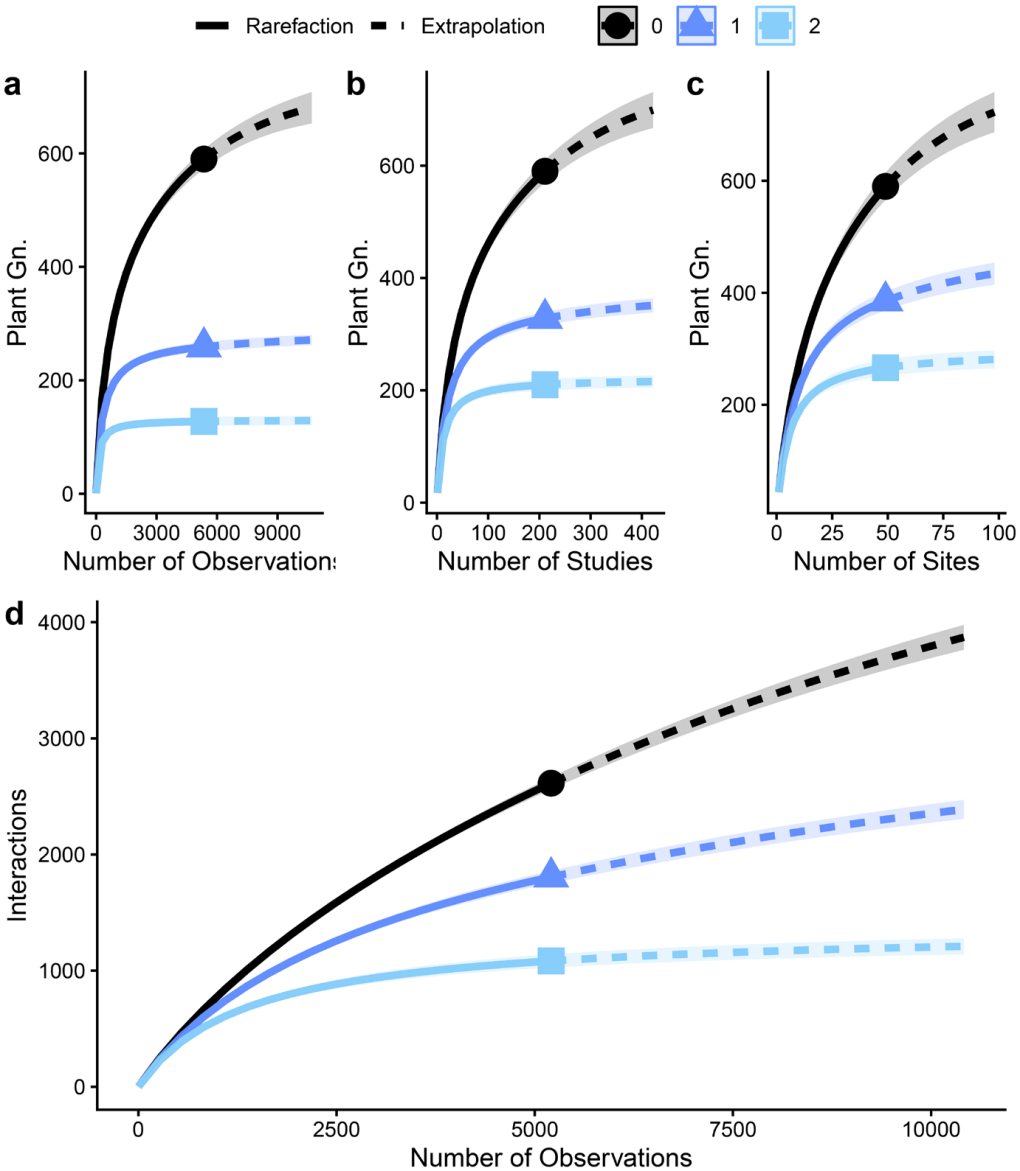
Rarefaction and extrapolation curves for the diversity of plant genera by the number of observations, number of studies, and number of sites, as well as lemur‐plant interactions by number of observations. Solid lines represent rarefaction and dashed lines represent extrapolation. Shaded areas represent 95% confidence intervals. Colors signify Hill numbers of order *q*: Richness (*q* = 0, black), exponential of Shannon's diversity (*q* = 1, blue) and inverse of Simpson's diversity (*q* = 2, light blue).

Plants were undersampled in the lemur dietary data literature. Observed richness of plant genera consumed by lemurs was 590 (extrapolated by number of observations = 668–775), the exponential of Shannon's diversity was 259 (269–288), and the inverse of Simpson's diversity was 127 (122–139; Figure 2a). Plant genus richness extrapolated by number of studies was 707–815 (Figure 2b), and by number of sites was 731–862 (Figure 2c). Therefore, plant genera sampling was limited relatively equally by number of observations, number of studies, and number of sites.

Up to 45% of dietary data between lemur species and plant genera may be missing from the literature. Observed interaction richness was 2614 (extrapolated by number of observations = 5177–5800), the exponential of Shannon's diversity was 1807 (3008–3258), and the inverse of Simpson's diversity was 1084 (1265–1471; Figure 2d). Interaction Simpson's diversity was better sampled than richness or Shannon's diversity, suggesting that rare interactions may have been especially undersampled. See Table S1 for full results.

### 3.2 Predictors of Dietary Richness

**FIGURE 3:**
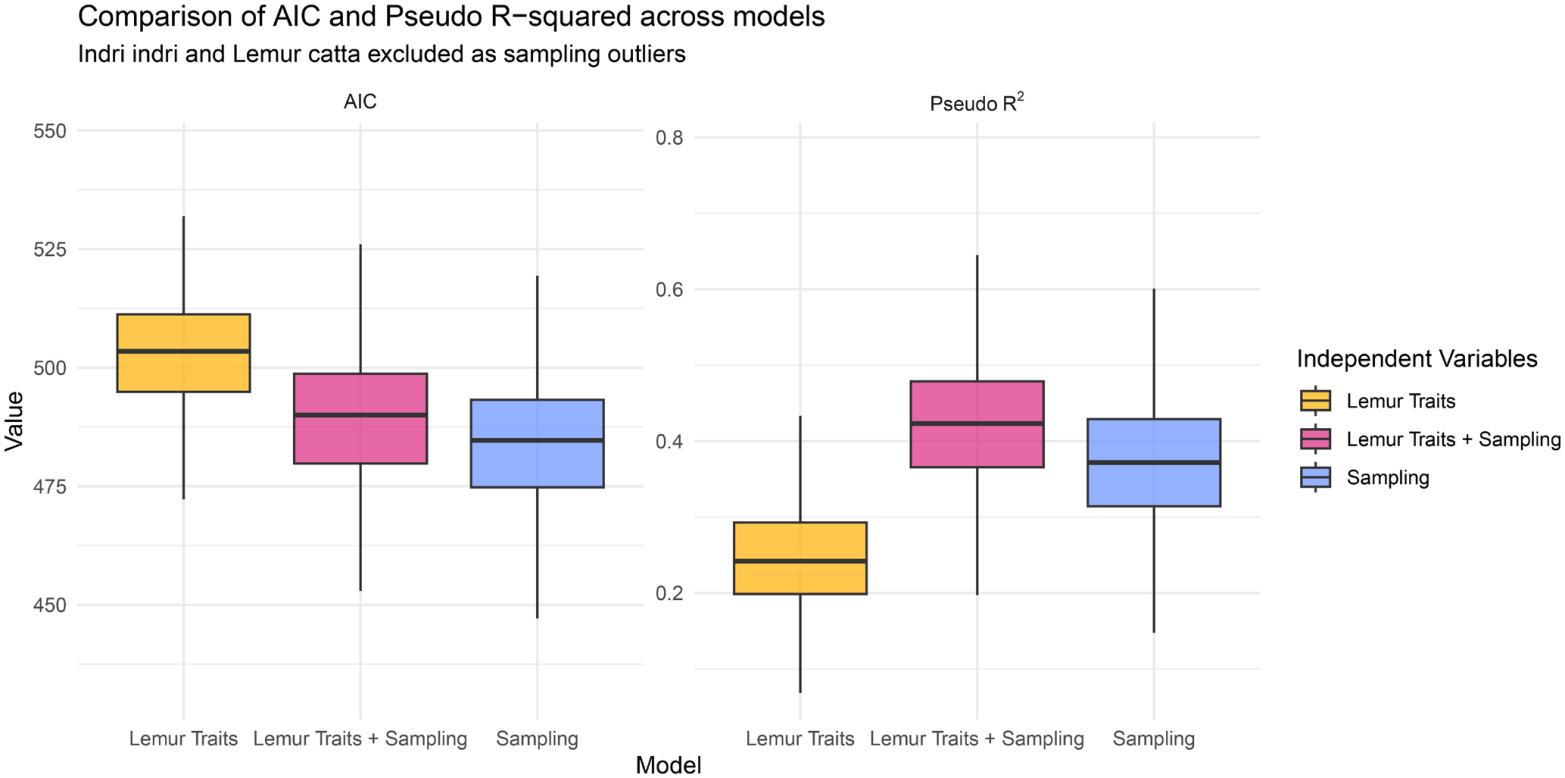
Comparison of goodness‐of‐fit of three negative binomial general linear models that attempt to predict dietary richness for each lemur species. On the left, the boxplots of a bootstrapped Akaike Information Criterion (AIC) over 1000 runs are displayed. The five lemur traits together gave a median AIC of 503.5, (IQR = 494.9–511.3), with empirical 95% CI of 476.7–525.4, while sampling effort and traits taken together performed better, with a lower AIC of 490.0, (IQR = 479.8–498.7, CI = 462.6–514.0). However, sampling effort alone yielded the best median AIC of 484.7, (IQR = 474.8–493.2; CI = 457.5–510.1). On the right, the boxplots of pseudo‐*R* ^2^ (also known as McFadden's *R* ^2^) are shown. Pseudo‐*R* ^2^ demonstrated a slightly different hierarchy of the goodness‐of‐fit: the model that includes lemur traits together with sampling effort as predictors performed the best, with the median of 0.423, IQR of 0.366–0.479 and CI of 0.270–0.586, but sampling effort alone still significantly outperformed the five lemur traits combined. The median bootstrapped value for the sampling effort model was 0.372 (IQR = 0.314–0.429, CI = 0.215–0.526), while the traits only model median pseudo‐R‐squared value was only 0.242 (IQR = 0.199–0.293, CI = 0.122–0.400).

**FIGURE 4:**
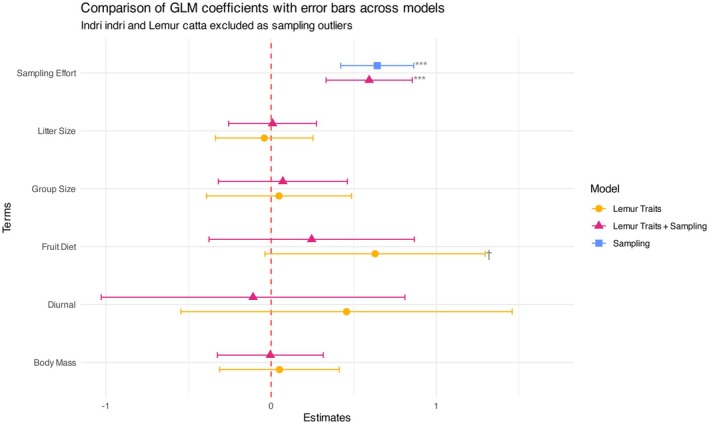
The predictor estimates with 95% CIs from original non‐bootstrapped models and corresponding *p*‐value significance levels of all three negative binomial general linear models discussed in Figure 3. Significance codes: ***p < 0.001, **p < 0.01, *p < 0.05, †*p* < 0.1; no symbol indicates *p* ≥ 0.1. When sampling effort was added as a predictor to the five lemur traits, the estimated effect of fruit diet reduced and lost the little significance it had. While no other traits were statistically significant in the lemur trait model, some estimate movement was also observed in the diurnality variable once sampling effort was added.

The model with only the sampling effort predictor tended to have the lowest AIC score (Figure 3a), indicating better model fit than the lemur trait models. Based on the pseudo‐*R* ^2^ method, the sampling effort model was slightly outperformed only by the model incorporating five lemur traits and sampling effort as predictors (Figure 3b). The lemur traits were not statistically significant predictors of dietary richness; sampling effort bore the entire explanatory weight (Figures 3 and 4). The greater pseudo‐*R* ^2^ score of the traits and sampling model may be attributed to the larger number of predictors included in the model.

With a wAIC of 0.989, the sampling effort model was approximately 99 times more likely to be the best approximating model within our candidate set compared to the lemur traits and sampling model (wAIC = 0.011). The model including only lemur traits received negligible support (wAIC = 2.00e‐05). Note that these results were robust to the inclusion of the outliers— *Indri indri* and *Lemur catta* (see Table S5).

The comparison of coefficients across the three models revealed a small shift in the influence of frugivory and diurnality variables (Figure 4, Tables S2–S4). When sampling effort was introduced as a predictor, it emerged as the most critical determinant of observed dietary richness, while lemur traits apart from frugivory were not significant predictors of lemur dietary richness (Figure 4).

### 3.3 Biased Prediction

**FIGURE 5:**
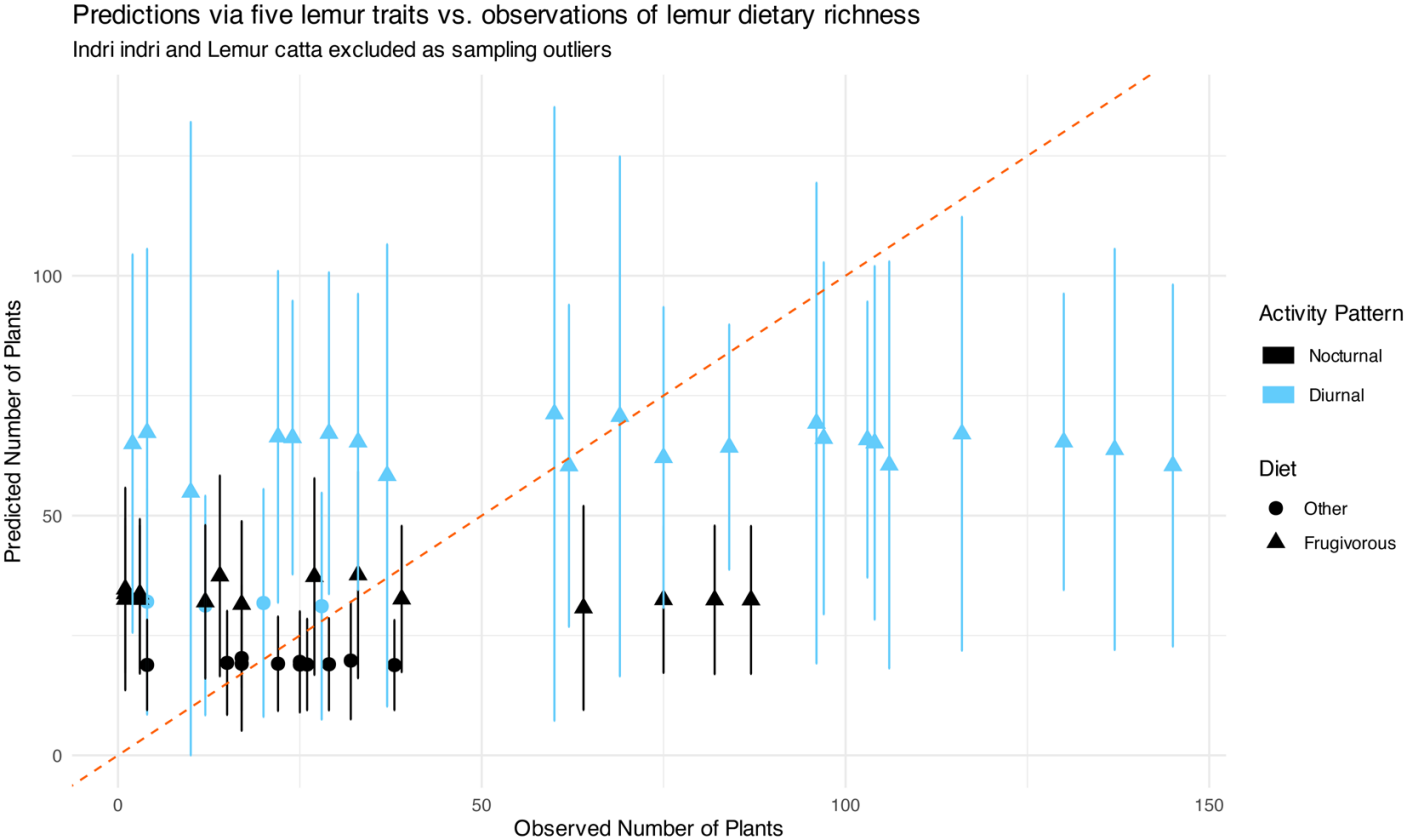
The predictions of lemur dietary richness with 95% error bars extracted from the negative binomial general linear model that only included functional traits (did not include sampling effort as a predictor), color‐coded by activity pattern and shape‐coded by diet. Lemurs with highest predicted dietary richness were all diurnal and frugivorous. Nocturnal and frugivorous lemurs with only four diurnal non‐frugivorous species comprise the second‐highest strata of predicted dietary richness. Lemurs with lowest predicted dietary richness were exclusively nocturnal and non‐frugivorous.

When we used only the five lemur traits to predict dietary richness, diet and activity pattern emerged as the only potential predictors with non‐zero median estimates (Figure 4). Comparing observed versus predicted dietary richness of the five‐trait model (Figure 5), the predicted values were stratified into three levels: the highest for diurnal frugivorous lemurs, an intermediate level primarily for nocturnal frugivorous lemurs, and the lowest for nocturnal non‐frugivorous species. Including sampling effort as a predictor minorly corrected this implicit stratified bias (see Figure S2 and coefficients in Figure 4).

## 4 Discussion

Overall, our results strongly indicate that sampling bias plays an outsized role in current lemur dietary data. Undersampling may result in missing up to 45% of dietary interactions between lemur species and plant genera (Figure 2d). Additionally, sampling effort remained the most important predictor of observed dietary behavior: the model incorporating solely sampling effort received disproportionately high support, as quantified by the Akaike model weights (wAIC). Specifically, upon exclusion of outliers, this model was approximately 99 times more likely than the model including both lemur traits and sampling as predictors. Even with outliers retained (see Figures S3 and S4 and Tables S5–S8), the sampling‐effort‐only model still garnered substantially greater support (wAIC = 0.35) compared to the five‐trait‐only model (wAIC = 0.02) (Table 1 and Table S5). The observed sampling bias may be predominantly attributable to the previously identified ecological field bias, which results in the disproportionate observation of diurnal over nocturnal and frugivorous over non‐frugivorous lemur species. These findings suggest there might exist causal mediation effects that require further analyses.

### 4.1 Sampling Effect

Sampling effort might mediate the effects of certain functional traits on dietary richness (Tables S15–S17). Consequently, failing to account for sampling effort leads to an inaccurate understanding of the biological role of functional traits in dietary diversity. The importance of lemur dietary guild (whether or not a species is a frugivore), and especially activity pattern (diurnal or nocturnal), was overestimated when sampling effort was excluded from our dietary richness model (Figure 4), highlighting that lemur diet research has been biased towards frugivorous, diurnal lemurs. Notably, our results from the negative binomial models suggest that dietary richness was not significantly related to whether a lemur was diurnal or nocturnal (Figure 4, Table 1). Although nocturnal lemurs are often underrepresented in ecological research compared to diurnal lemurs, they exert important ecological functions such as seed dispersal (Ramananjato 2024; Ramananjato et al. 2020). Unlike research conducted with neotropical primates (Hawes and Peres 2014), body mass was not related to dietary richness (Figure 4).

### 4.2 Phylogenetic Insight

Biological traits often display phylogenetic signal (Cadotte et al. 2019). It is therefore challenging to disentangle whether any observed trait‐based relationships are caused by the traits themselves or shared evolutionary history and phylogenetic conservatism. Many common frequentist approaches that account for phylogenetic non‐independence of species data do not support negative binomial or Poisson distributions (e.g., Orme et al. 2023). Therefore, we accounted for lemur phylogenetic relationships using MCMCglmm (Hadfield 2010) (Figures S9 and S10). These models generally supported the main analysis results. While mean Pagel's *λ* values suggest that there was moderate phylogenetic signal in lemur dietary richness, high uncertainty means that we cannot rule out no phylogenetic signal.

Considering phylogenetic effects in dietary studies may reveal biologically important traits. For example, our phylogenetic analysis accounting for sampling effort suggested that group size may be positively predictive of lemur dietary (Figure S9; marginally significant). Sociality in mammals improves foraging efficiency and interspecific competitive ability (Silk 2007). Therefore, when desirable food resources are patchy across the environment, lemurs in larger groups may be able to better access and monopolize those resources. Indeed, lemur group size predicts social cognition (MacLean et al. 2013), and *Lemur catta* from larger group sizes gain foraging advantages through access to favorable habitat (Pride 2005). There are, however, competitive trade‐offs between group sizes. For example, lemurs in large groups exhibit high stress levels when food resources are scarce, suggesting that intraspecific competition for food inherent to larger group sizes may also have negative consequences for fitness (Pride et al. 2006). To improve understanding of the relationship between dietary specialization and life history traits such as group size, we advocate for future research that further investigates phylogenetic conservatism in dietary ecology.

### 4.3 Variable Mediation Effects

Together with other ecological studies (e.g., Allison and Destefano 2006; Razafindratsima et al. 2022), our analyses indicate that traits such as frugivory and diurnality bias sampling. Consequently, when treated as a predictor of lemur dietary richness, sampling effort may act as a mediating confounder of these traits on true dietary behavior. We conducted several mediation analyses with available software, but each comes with significant caveats. Since the widely used *mediate* package does not support negative binomial models, we modeled effects of mediation via Poisson models. While results support our mediation hypothesis, they do not account for severe overdispersion of data, so the results are likely to be overconfident (see Data 5.1). The *maczic* package allowed us to deploy negative binomial mediation and outcome models by providing their intercepts and coefficients, but its priors are standard normal distributions by default and are very non‐informative. While the choice of the prior default is understandable, the inability to build more informative priors based on known lemur trait values introduces very wide confidence intervals and large uncertainty. We observed only 4%–5% of simulations where the mediation effect was detected, but we believe that this is likely due to prior inflexibility and bias (see Data 5.2). It is out of the scope of this paper to build robust software that can analyze mediation effects for strongly overdispersed data with a variety of covariates with different distributions, and we recommend further investigation into the mediation phenomenon of sampling effort. Note that our models involve assumptions and results interpretation should be treated with caution. However, the different statistical analyses considered here consistently point towards sampling effort playing a role in mediating how functional traits—especially frugivory—predict dietary richness.

### 4.4 Future Directions and Conservation Implications

Bipartite lemur–plant networks help us explore and analyze the eco‐evolutionary mechanisms and ecological consequences of trophic interactions (DeSisto and Herrera 2022; Tonos et al. 2024). Characterizing sources of bias is a critical step forward to improving network‐level metrics, but our analysis does not identify which links are missing. Future research on lemur–plant interactions should therefore employ new link prediction techniques that robustly account for sampling bias (e.g., Papadogeorgou et al. 2023; Kampe et al. 2025). Beyond addressing spatial, taxonomic, and trait‐based biases, it is equally important to recognize the temporal biases pervasive in biodiversity data (Daru et al. 2018). Investigating phenological biases in dietary data therefore represents a crucial step forward. By systematically accounting for various types of sampling bias, we will gain deeper insights into the ecological roles of species, shedding light on the fundamental mechanisms driving ecological interactions in tropical ecosystems.

A biased understanding of dietary specialization may limit effective conservation. For example, while DeSisto and Herrera (2022) accounted for sampling effort to identify the potential consequences of plant extinctions for lemur populations, additional dietary data would likely improve these estimations. Gaps in dietary data may prevent a more complete understanding of other conservation concerns on lemur foraging behavior and forest ecosystem functioning. For example, lemurs are known to use invasive species for habitat (Eppley et al. 2015), as well as feed on and disperse invasive plants (DeSisto et al. 2020). Lemur–plant interaction data are disproportionately concentrated in specific sites and species (Steffens 2020; Tonos et al. 2024). For instance, although Madagascar boasts 98 terrestrial protected areas, lemur dietary research has predominantly focused on Ranomafana National Park, where established infrastructure like the Centre ValBio facilitates research (Steffens 2020). Expanding research field stations to additional locations could enhance large‐scale biodiversity studies while improving conservation efforts (Eppley et al. 2024). The rarefaction and extrapolation curves (Figure 2c) suggest we are far from adequately sampling lemur diet across all possible sites. Therefore, to improve the completeness of lemur dietary datasets, we believe it is crucial to prioritize empirical studies in underrepresented sites. Community‐managed protected areas, which are often overlooked in the literature, are promising locations for current future lemur‐plant interaction research (e.g., DeSisto, Bezaralahy, et al. (2025)). Moreover, evidence from other regions, such as studies on diverse taxonomic groups in Brazil, highlights the widespread issue of spatially biased sampling in biodiversity research (Oliveira et al. 2016). Addressing spatial sampling bias is particularly important for dietary data, where biases may compound across trophic levels. Underestimated or overlooked feeding areas in conservation management plans may lead to the insufficient protection of habitats that provide essential food resources to endangered species such as lemurs.

## 5 Conclusions

Our study underscores the pervasive influence of sampling bias on understanding lemur dietary specialization and trophic interactions. Building on rarefaction and extrapolation diversity analyses with trait‐based modeling of dietary richness, we find that sampling effort could have some mediating effect on activity patterns and diet, resulting in an improper or insufficient estimation of the predictive power of these functional traits for explaining dietary richness. Sampling bias may also mask the significance of other functional traits that have an ecologically significant relationship to dietary richness. These findings highlight the critical need for comprehensive data collection strategies that address taxonomic, geographic, and temporal biases. Sampling nocturnal and non‐frugivorous lemurs, expanding research efforts to underrepresented sites, particularly community‐managed protected areas, and further investigating potential phenological bias will improve data completeness and the ecological insights derived from it. Such approaches are essential not only for accurately estimating the effects of functional traits on dietary richness, but also for advancing effective sampling methodologies across tropical ecosystems.

## Author Contributions

**Anna Vasenina:** conceptualization (equal), formal analysis (lead), methodology (equal), visualization (equal), writing – original draft (equal), writing – review and editing (equal). **Camille M. M. DeSisto:** conceptualization (equal), data curation (lead), formal analysis (supporting), methodology (equal), visualization (equal), writing – original draft (equal), writing – review and editing (equal). **Peter J. Mucha:** conceptualization (supporting), formal analysis (supporting), funding acquisition (lead), methodology (equal), supervision (lead), writing – review and editing (equal).

## Funding

This work was supported by the National Science Foundation EEID, DEB‐2308460.

## Disclosure

*Software Tools*—Sampling completeness: R package iNEXT version 3.0.1 (Chao et al. 2014) and iNEXT.link version 1.0.0 (Hsieh et al. 2016). Causal mediation analysis with the mediate function: R package mediation version 4.5.0 on CRAN (Tingley et al. 2014). Negative binomial mediation analysis with the maczic_power function: R package maczic version 1.1.0 on CRAN (Chang et al. 2018). PCA With prcomp: R package stats version 4.4.1 on CRAN (R Foundation for Statistical Computing 2025). Negative Binomial GLM Models and AIC Scores with glm.nb function: R package MASS version 7.3‐24 60.2 on CRAN (Ripley 2007).

## Conflicts of Interest

The authors declare no conflicts of interest.

## Supporting information


**Data S1:** ece372765‐sup‐0001‐Supinfo.pdf.

## Data Availability

Data and code are published on a public repository: https://zenodo.org/records/17725395.
