# Supplementary material for: Untangling Sampling Bias From Lemur Dietary Specialization
Source: Ecol Evol. 2026 Jan 4;16(1):e72765. doi: 10.1002/ece3.72765 (PMC12765593; doi:10.1002/ece3.72765)
Supplement: Supplementary file 1 — Data S1: ece372765‐sup‐0001‐Supinfo.pdf. [file ECE3-16-e72765-s001.pdf]

Supplementary Information: Untangling sampling bias from lemur dietary richness

December 2, 2025

1 Principal Component Analysis

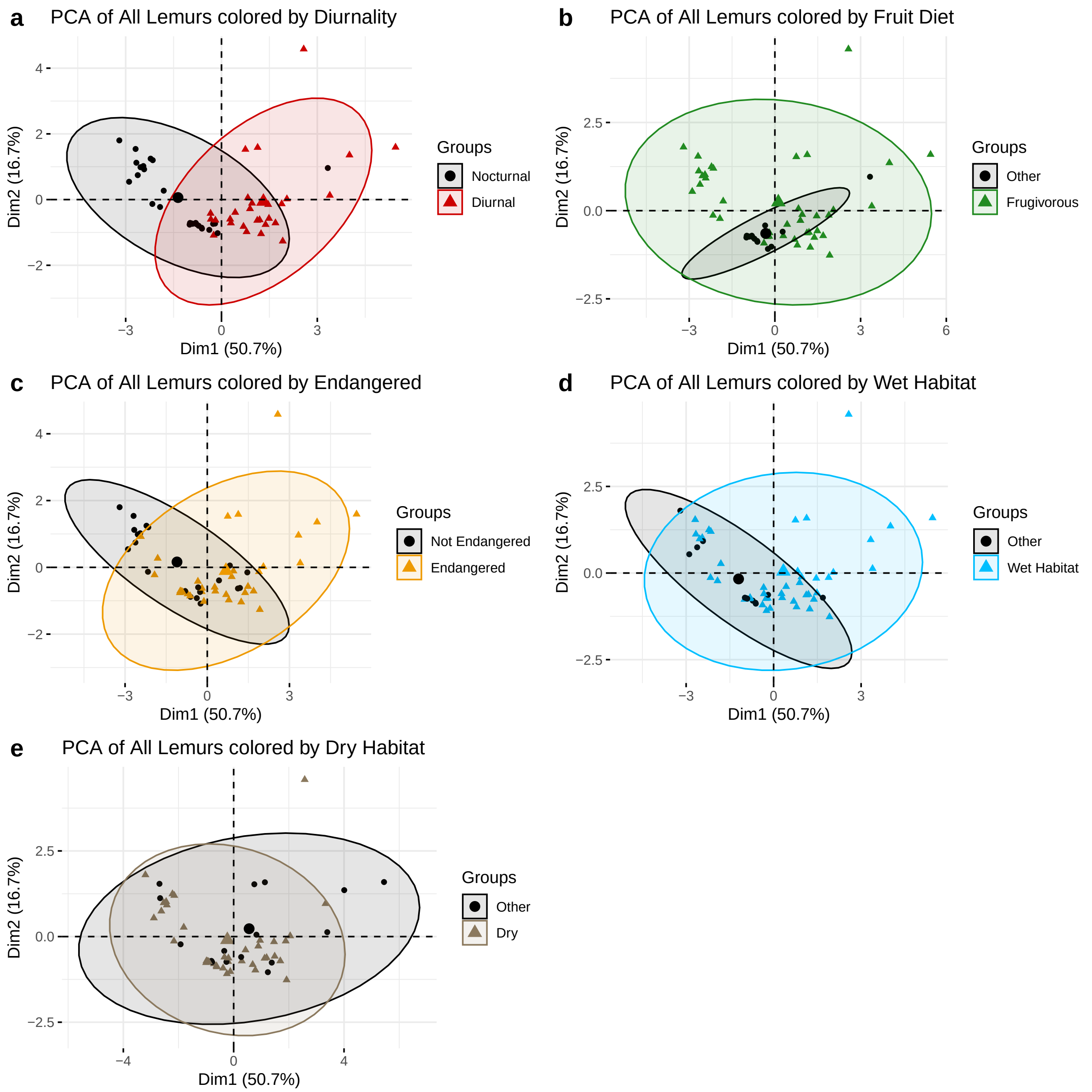

Supplementary Figure 1: PCA of lemurs colored by different binary variables: a) activity pattern, b) diet, c) endangered status, d) whether they live in wet habitat, e) whether they live in dry habitat. The large circle and large triangle in each figure designate the average PCA coordinate value for each of the two groups. Panel (a) shows distinct clustering into two groups: nocturnal and diurnal lemurs. Panel (b) shows strong clustering of non-frugivorous lemur species. Other binary traits do not exhibit clustering in PCA coordinates and, thus, were excluded from the list of predictors in the regression models that we consider.

2 Sampling Completeness

Type	Index	Observed	Estimated	LCL	UCL
Interaction	Richness	2614	5489	5177	5800
	Shannon's diversity	1807	3133	3008	3258
	Simpson's diversity	1084	1368	1265	1471
Plant Genera, Observations	Richness	590	721	668	775
	Shannon's diversity	259	279	269	288
	Simpson's diversity	127	131	122	139
Plant Genera, Studies	Richness	590	761	707	815
	Shannon's diversity	328	367	354	380
	Simpson's diversity	210	222	211	233
Plant Genera, Sites	Richness	590	797	731	862
	Shannon's diversity	385	474	455	493
	Simpson's diversity	266	304	284	323

Supplementary Table 1: Results of the rarefaction and extrapolation analyses, including the lower and upper confidence limits (LCL and UCL). Values are rounded to the nearest integer.

3 Predictors of Dietary Richness

3.1 Outliers

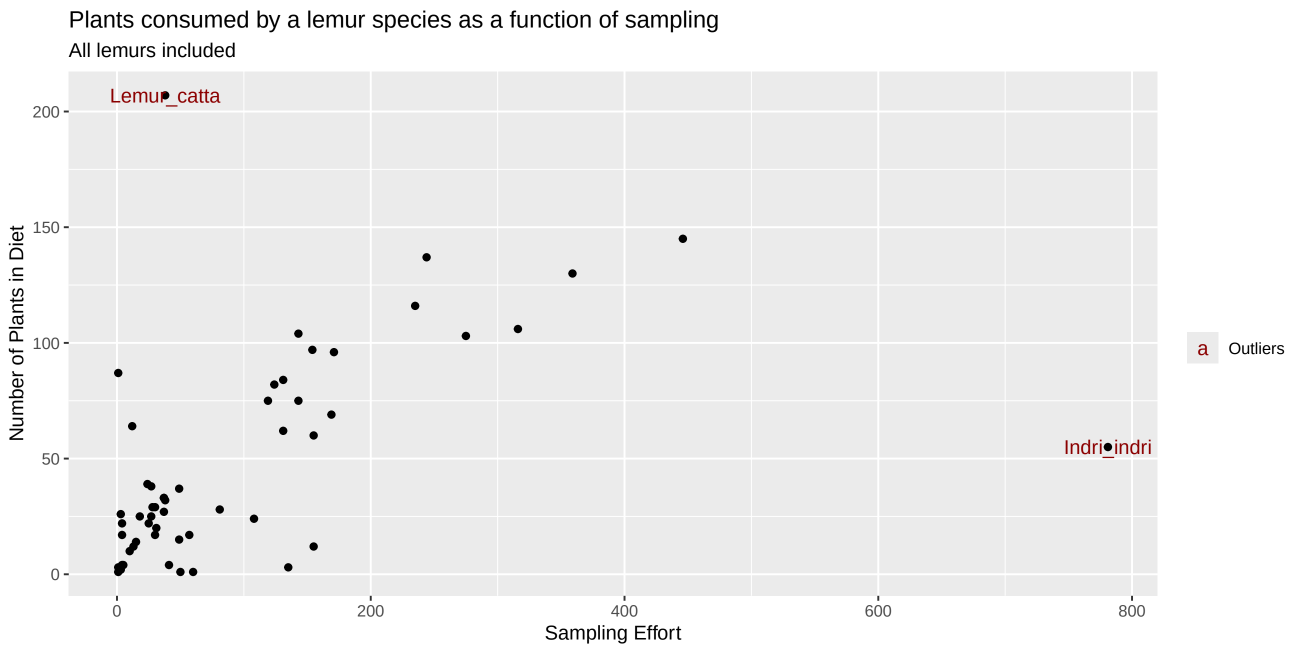

Supplementary Figure 2: The species *Indri indri* are of specific interest to ecologists and are substantially oversampled compared to all other species. Studies of *Lemur catta* may be fewer in number but result in high dietary richness if these studies tend to be more long-term, involve focal follows, and/or collect information from large groups.

3.2 Negative binomial coefficient estimates (outliers excluded)

Coefficient estimate results for original non-bootstrapped negative binomial linear models, used to generate Figures 3 and 4 and Table 1 in the main text.

	**Estimate**	**Std. Error**	**z value**	**Pr(> $\|z\|$)**
(Intercept)	2.98852	0.38467	7.769	7.91e-15 ***
Body Mass	0.05072	0.18478	0.274	0.7837
Group Size	0.04753	0.22396	0.212	0.8319
Litter Size	-0.04138	0.15046	-0.275	0.7833
Fruit Diet	0.62946	0.34025	1.850	0.0643 .
Diurnal	0.45649	0.51156	0.892	0.3722

Supplementary Table 2: The negative binomial linear model coefficients, standard errors, z-values, and p-values of independent variables across 53 lemurs in the five-trait model.

	**Estimate**	**Std. Error**	**z value**	**Pr(> $\|z\|$)**
(Intercept)	3.438526	0.363382	9.463	<2e-16 ***
Body Mass	-0.004883	0.163533	-0.030	0.976
Group Size	0.070869	0.199384	0.355	0.722
Litter Size	0.008642	0.135574	0.064	0.949
Fruit Diet	0.245393	0.317000	0.774	0.439
Diurnal	-0.109134	0.469004	-0.233	0.816
Sampling Effort	0.593442	0.133062	4.460	8.2e-06 ***

Supplementary Table 3: The negative binomial linear model coefficients, standard errors, z-values, and p-values of independent variables across 53 lemurs in the five-trait and sampling model.

	**Estimate**	**Std. Error**	**z value**	**Pr(> $\|z\|$)**
(Intercept)	3.5678	0.1130	31.576	< 2e-16 ***
Sampling Effort	0.6423	0.1127	5.698	1.22e-08 ***

Supplementary Table 4: The negative binomial linear model coefficients, standard errors, z-values, and p-values of independent variables across 53 lemurs in the sampling effort only model.

3.3 Negative binomial: all lemurs included

3.3.1 Goodness-of-fit measures

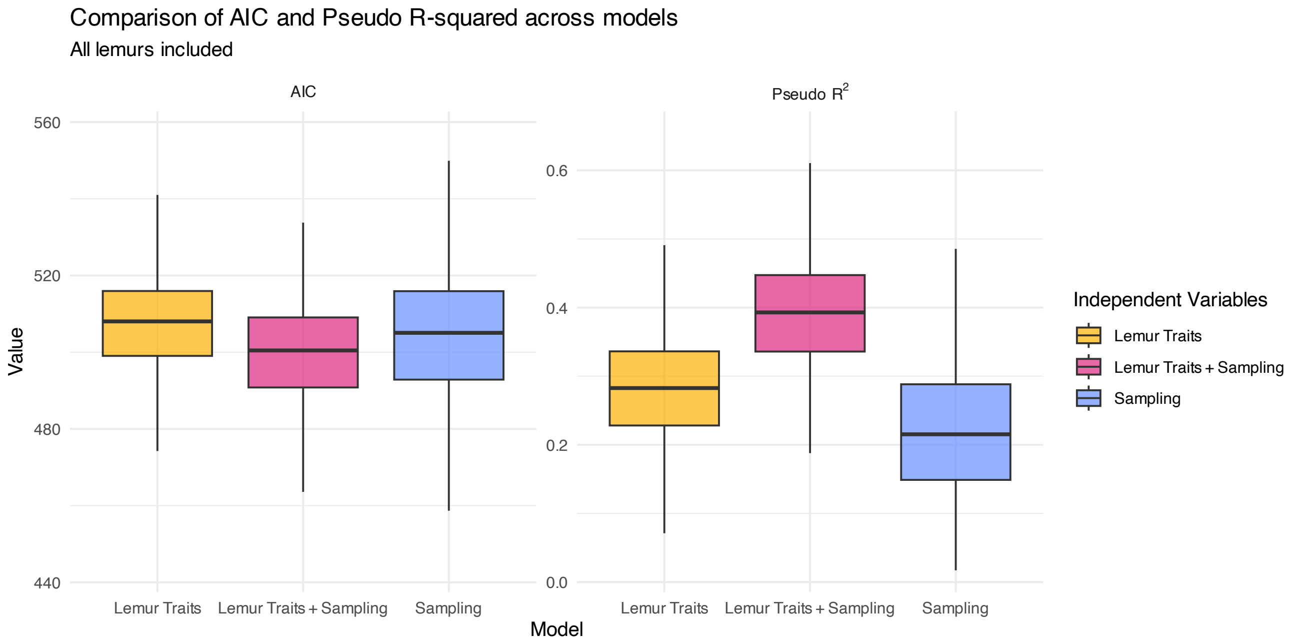

Supplementary Figure 3: Comparison of goodness-of-fit of three negative binomial general linear models, including all lemurs, that attempt to predict dietary richness for each lemur species. On the left the IQR of Akaike Information Criterion (AIC) is displayed. On the right the IQR of pseudo-R-squared (also known as McFadden's R-squared) is shown. According to the AIC measures, all three models perform comparably. Pseudo-R-squared ranks the lemur traits and sampling model as the best, then traits only as predictors second, and sampling alone last.

For more rigorous model comparison of the three negative binomial models that include all lemur data, we additionally analyzed ΔAIC and wAIC, similarly to the models discussed in the main text. Traits and sampling remains the most significant model, with an Akaike weight of 0.632, meaning 63.2% of the Akaike-model-weighted evidence favors this model among those considered (Table 5). Sampling alone comes in a close second, with 34.5% support. While the AIC of the lemur-traits-only model is comparable, it receives negligible support (2.4%) in terms of Akaike weights.

Model	AIC	ΔAIC	wAIC
Sampling	526.5	1.210	0.345
Lemur Traits + Sampling	525.3	0.000	0.632
Lemur Traits	531.9	6.576	0.024

Supplementary Table 5: Model selection and relative support: comparison of lemur dietary richness models with all lemurs included. ΔAIC and Akaike weights for a model set consisting of three non-bootstrapped, negative binomial regression models with differing predictor sets, initialized with a fixed dispersion theta value of 1.205 (see section 2.2.3).

3.3.2 Coefficient estimates

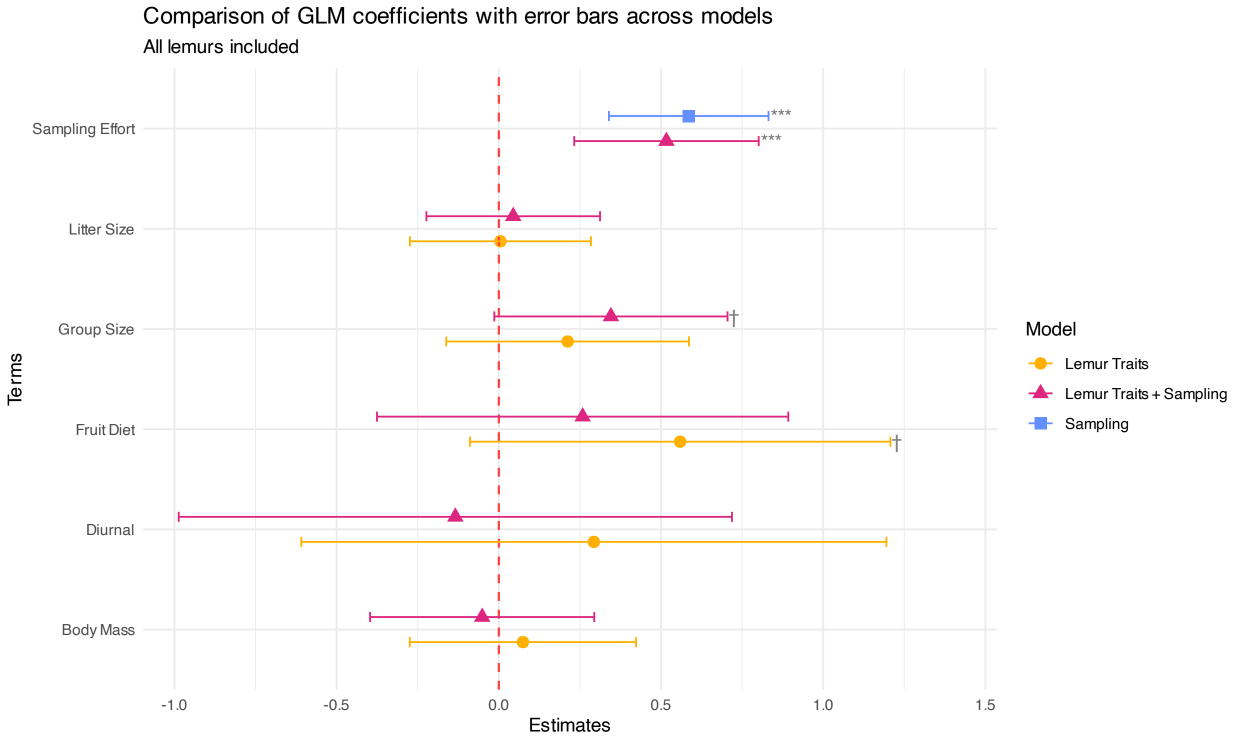

Supplementary Figure 4: The predictors' estimates with 95% CIs and corresponding p-values of all three negative binomial general linear models discussed in Supplementary Figure 3. When sampling effort is added as a predictor to the five lemur traits, diet behavior (frugivorous or not) loses all significance, and the activity pattern coefficient estimate moves in the same direction, towards zero (and possibly negative). On the other hand, with sampling effort included, group size becomes a statistically significant predictor of lemur dietary richness. Significance codes: *** $p < 0.001$, ** $p < 0.01$, * $p < 0.05$, † $p < 0.1$; no symbol indicates $p \geq 0.1$.

	**Estimate**	**Std. Error**	**z value**	**Pr(> |z|)**
(Intercept)	3.150378	0.360796	8.732	<2e-16 ***
Body Mass	0.074284	0.177944	0.417	0.6763
Group Size	0.212022	0.190926	1.110	0.2668
Litter Size	0.004887	0.142391	0.034	0.9726
Fruit Diet	0.559051	0.330653	1.691	0.0909 .
Diurnal	0.292933	0.460166	0.637	0.5244

Supplementary Table 6: The negative binomial linear model coefficients, standard errors, z-values, and p-values of independent variables across all 55 lemurs in the five-trait model.

	**Estimate**	**Std. Error**	**z value**	**Pr(> |z|)**
(Intercept)	3.52682	0.35178	10.026	<2e-16 ***
Body Mass	-0.05133	0.17632	-0.291	0.770978
Group Size	0.34553	0.18342	1.884	0.059590 .
Litter Size	0.04433	0.13656	0.325	0.745455
Fruit Diet	0.25839	0.32347	0.799	0.424394
Diurnal	-0.13413	0.43510	-0.308	0.757871
Sampling Effort	0.51678	0.14506	3.562	0.000367 ***

Supplementary Table 7: The negative binomial linear model coefficients, standard errors, z-values, and p-values of independent variables across all 55 lemurs in the five-trait and sampling model.

	**Estimate**	**Std. Error**	**z value**	**Pr(> |z|)**
(Intercept)	3.7272	0.1256	29.678	< 2e-16 ***
Sampling Effort	0.5855	0.1255	4.664	3.11e-06 ***

Supplementary Table 8: The negative binomial linear model coefficients, standard errors, z-values, and p-values of independent variables across all 55 lemurs in the sampling-effort-only model.

3.4 Poisson model goodness-of-fit with outliers excluded

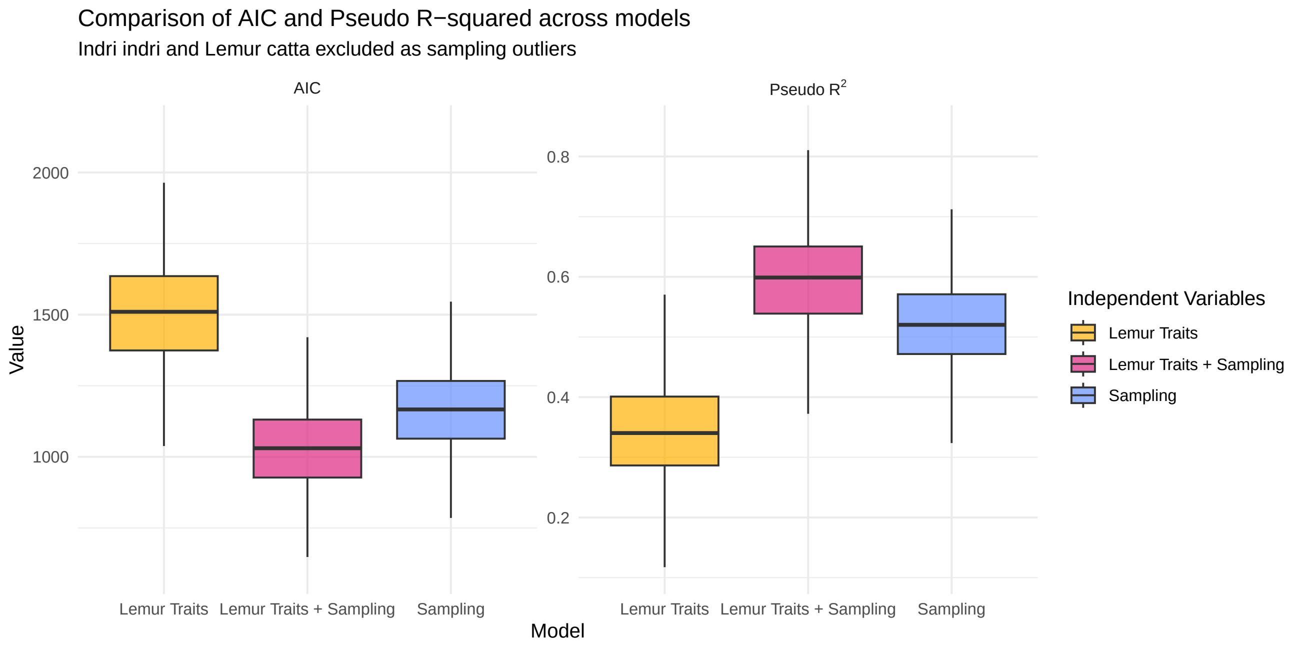

Supplementary Figure 5: Comparison of goodness-of-fit of three Poisson general linear models that attempt to predict dietary richness for each lemur species. On the left the IQR of Akaike Information Criterion (AIC) is displayed. On the right the IQR of pseudo-R-squared (also known as McFadden's R-squared) is shown. The model that includes lemur traits together with sampling effort as predictors performs the best, while sampling effort alone outperforms the five lemur traits combined.

The five lemur traits together gave a bootstrapped median AIC over 1000 runs of 1510.1, with empirical 95% CI of 1152.5–1878.3, while sampling effort alone yielded a better, lower median AIC of 1166.8 (887.8–1486.2; Supplementary Fig. 5). Sampling effort and traits taken together performed the best, with an AIC of 1030.4 (758.0–1335.4).

Pseudo-R-squared demonstrated a similar hierarchy of the goodness-of-fit, with sampling effort alone outperforming the set of five lemur traits for predicting lemur dietary richness. The median bootstrapped value for sampling effort alone was 0.520 (CI of 0.367–0.655; Supplementary Fig. 5), while the traits model median Pseudo-R-squared value was only 0.340 (0.176–0.521). As expected, sampling and traits together was the best model, with the highest pseudo-R-squared of 0.599 (0.436–0.746).

3.4.1 Coefficient estimates

	**Estimate**	**Std. Error**	**z value**	**Pr($>|z|$)**
(Intercept)	2.74094	0.07738	35.424	<2e-16 ***
Body Mass	-0.02270	0.02855	-0.795	0.42650
Group Size	-0.01823	0.03176	-0.574	0.56596
Litter Size	-0.07977	0.02418	-3.299	0.00097 ***
Fruit Diet	0.77445	0.06757	11.461	<2e-16 ***
Diurnal	0.71578	0.08491	8.430	<2e-16 ***

Supplementary Table 9: The Poisson linear model coefficients, standard errors, z-values, and p-values of independent variables across 53 lemurs in the five-trait model.

	**Estimate**	**Std. Error**	**z value**	**Pr($>|z|$)**
(Intercept)	3.35451	0.08122	41.300	<2e-16 ***
Body Mass	-0.02461	0.03170	-0.776	0.438
Group Size	0.15854	0.03295	4.811	1.50e-06 ***
Litter Size	-0.02148	0.02874	-0.747	0.455
Fruit Diet	0.31692	0.07406	4.279	1.88e-05 ***
Diurnal	-0.01589	0.09875	-0.161	0.872
Sampling Effort	0.46241	0.01992	23.211	<2e-16 ***

Supplementary Table 10: The Poisson linear model coefficients, standard errors, z-values, and p-values of independent variables across 53 lemurs in the model with five lemur traits and sampling effort as predictors.

	**Estimate**	**Std. Error**	**z value**	**Pr($>|z|$)**
(Intercept)	3.61310	0.02346	154.03	<2e-16 ***
Sampling Effort	0.50647	0.01466	34.54	<2e-16 ***

Supplementary Table 11: The Poisson linear model coefficients, standard errors, z-values, and p-values of independent variables across 53 lemurs in the sampling effort model.

The comparison of coefficients across the three models revealed a substantial shift in the influence of frugivory and diurnality variables. When sampling effort was introduced as a predictor, it emerged as the most critical determinant of dietary richness (Supplementary Fig. 6). Concurrently, the coefficient for diurnality dropped to nearly zero (and with CI of nearly equal range on both sides of zero), suggesting that lemur activity patterns were overestimated and that diurnal species were likely overrepresented in the data. While frugivory

retained a positive effect, its impact diminished to less than one-half of its original weight obtained without including sampling effort. Notice that, with the overestimation of both diurnality and frugivory in the lemur-traits-only model, the influence of lemur group size was minimal; in contrast, and importantly, with the inclusion of sampling effort, group size became a significant predictor.

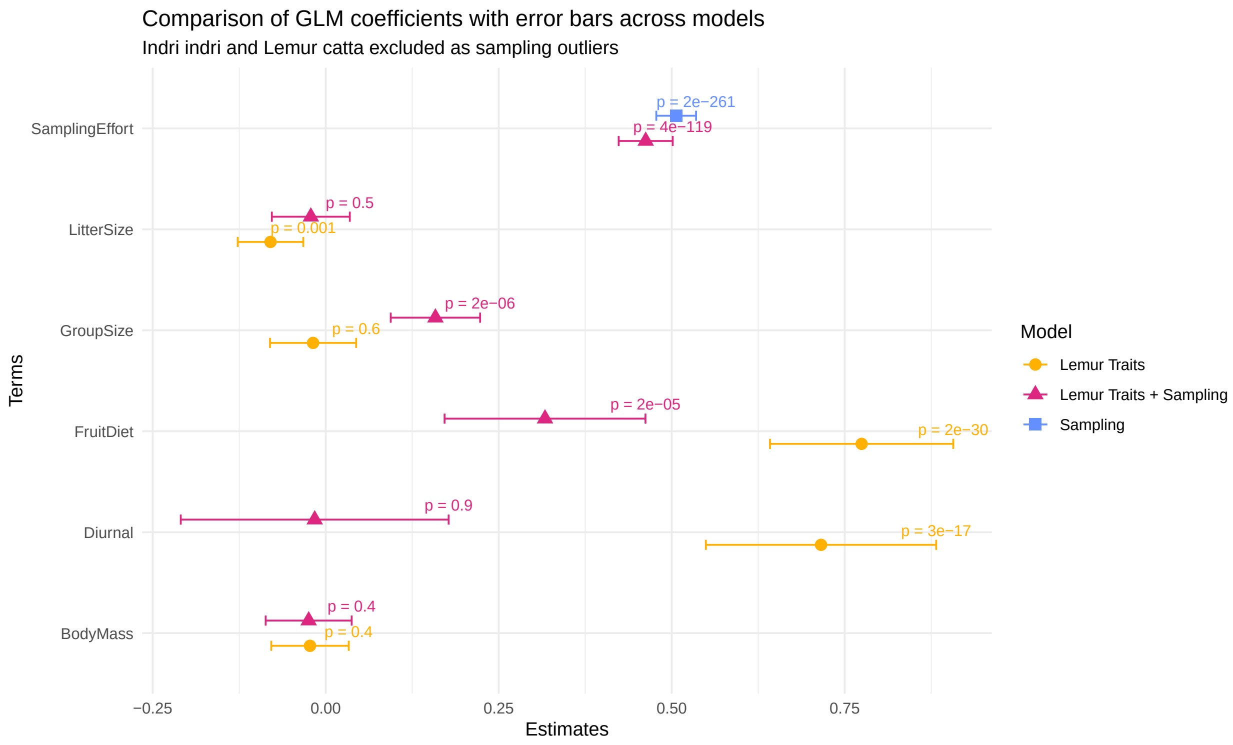

Supplementary Figure 6: The predictors' estimates with 95% CIs and corresponding p-values of all three Poisson general linear models discussed in Supplementary Figure 5. When sampling effort is added as a predictor to the five lemur traits, activity pattern (diurnal or nocturnal) loses all significance, while the estimated effect of diet reduces (but maintains significance). On the other hand, with sampling effort included, group size becomes a statistically significant predictor of dietary richness.

3.5 Poisson model goodness-of-fit with all lemurs included

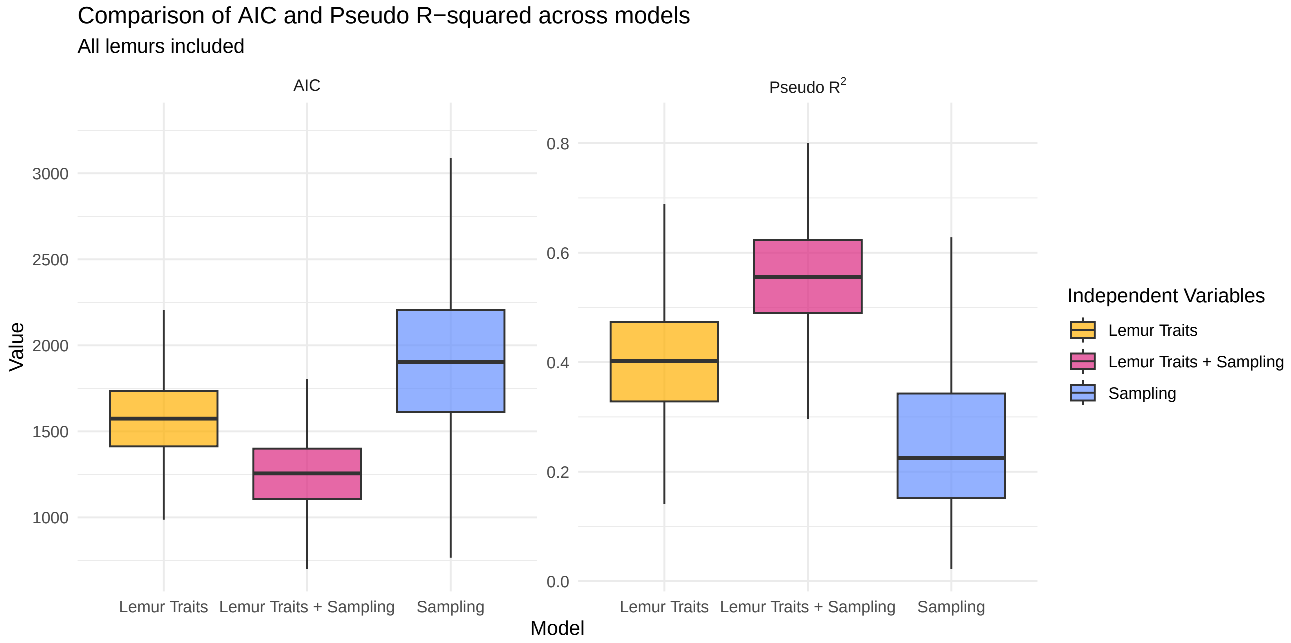

Supplementary Figure 7: Comparison of goodness-of-fit of three Poisson general linear models that attempt to predict dietary richness for each lemur species. *All* lemur species are included (to contrast with the models in the previous subsection). On the left the IQR of Akaike Information Criterion (AIC) is displayed. On the right, the IQR of pseudo-R-squared (also known as McFadden's R-squared) is shown. The model that includes lemur traits and sampling effort as predictors performs the best. However, lemur traits altogether outperform sampling effort on its own. While the predictive power of sampling effort is diminished due to its inability to explain some outliers, sampling effort still significantly improves the prediction of the five trait model and remains an important predictor in a larger model.

3.5.1 Coefficient estimates

	**Estimate**	**Std. Error**	z **value**	**Pr(**$>|z|$**)**
(Intercept)	3.01643	0.07212	41.825	<2e-16 ***
Body Mass	0.01658	0.02764	0.600	0.549
Group Size	0.23409	0.02266	10.331	<2e-16 ***
Litter Size	-0.03405	0.02260	-1.506	0.132
Fruit Diet	0.66618	0.06702	9.941	<2e-16 ***
Diurnal	0.37235	0.07563	4.924	8.5e-07 ***

Supplementary Table 12: The Poisson linear model coefficients, standard errors, z-values, and p-values of independent variables across 55 lemurs in the five-trait model.

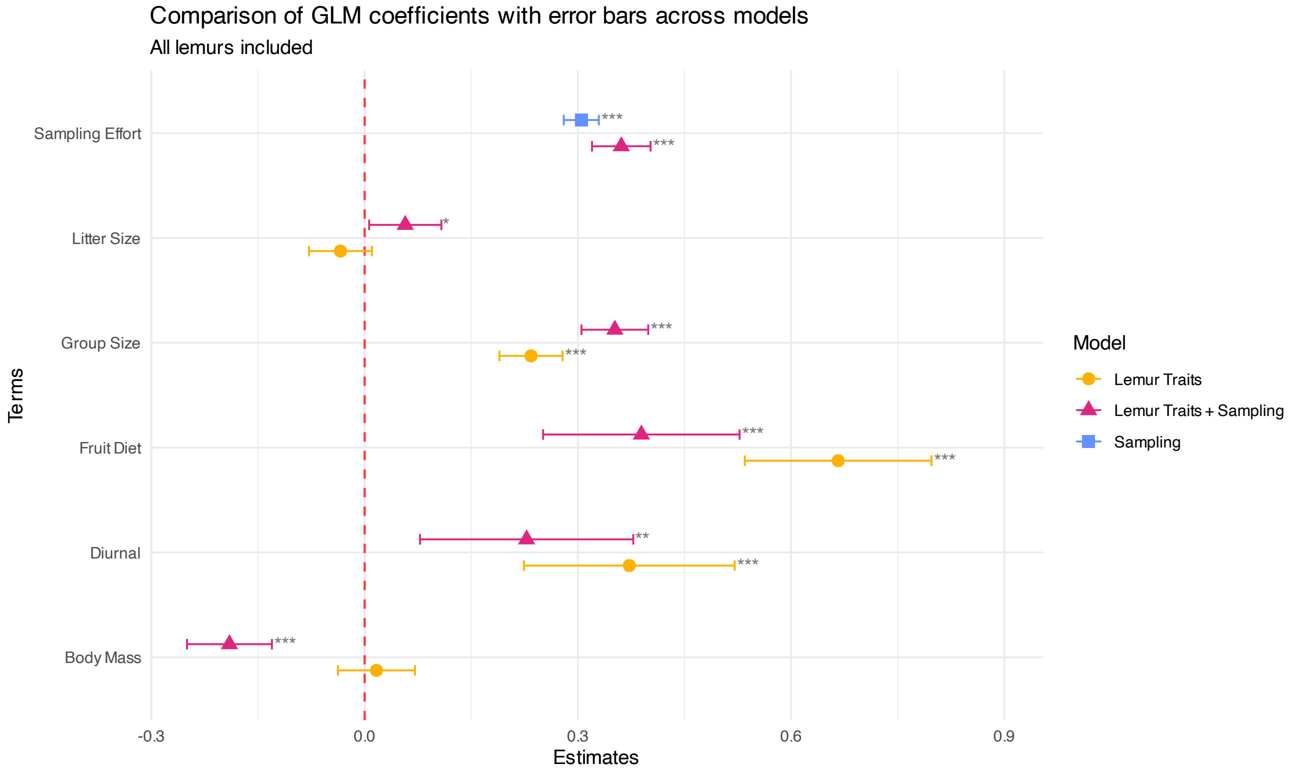

Supplementary Figure 8: The predictors' estimates with 95% CIs and corresponding p-values of all three Poisson general linear models discussed in Supplementary Figure 7.

	**Estimate**	**Std. Error**	z **value**	**Pr**$(>\|z\|)$
(Intercept)	3.25039	0.07320	44.402	<2e-16 ***
Body Mass	-0.19023	0.03048	-6.240	<4.37e-10 ***
Group Size	0.35200	0.02397	14.683	<2e-16 ***
Litter Size	0.05710	0.02592	2.202	0.02763 *
Fruit Diet	0.38932	0.07053	5.520	3.39e-08 ***
Diurnal	0.22785	0.07655	2.976	0.00292 **
Sampling Effort	0.36106	0.02102	17.180	<2e-16 ***

Supplementary Table 13: The Poisson linear model coefficients, standard errors, z-values, and p-values of independent variables across 55 lemurs in the model with five lemur traits and sampling effort as predictors.

	**Estimate**	**Std. Error**	z **value**	**Pr**$(>\|z\|)$
(Intercept)	3.78633	0.02061	183.72	<2e-16 ***
Sampling Effort	0.30481	0.01258	24.23	<2e-16 ***

Supplementary Table 14: The Poisson linear model coefficients, standard errors, z-values, and p-values of independent variables across 55 lemurs in the sampling effort model.

4 Phylogenetic Model

To account for phylogenetic non-independence of our data, we built Poisson mixed effects models using the same specifications as those in the main text (excluding *I. indri* and *L. catta*), but in a Bayesian framework using the `MCMCglmm` package [Hadfield, 2010]. Additionally, we accounted for phylogeny by accounting for the inverse phylogenetic similarity of lemur species in the models. In line with the trait imputation, we constructed the phylogenetic tree of the 55 lemur species used in trait imputations using the `U.PhyloMaker` package [Jin and Qian, 2023] and Upham et al.'s 2019 mammal megatree [Upham et al., 2019]. We set very weakly informative prior parameters to allow a wide range of possible variance values; we fixed the residual variance at 1 and the degrees of freedom for the inverse-Wishart distribution at 0.002. For each model, we ran the MCMC (Markov Chain Monte Carlo) algorithm for 50,000 iterations, discarded the first 2,000 iterations as burn-in, and did not apply. We assessed model convergence using trait plots. We also calculated Pagel's λ, a measure of phylogenetic signal.

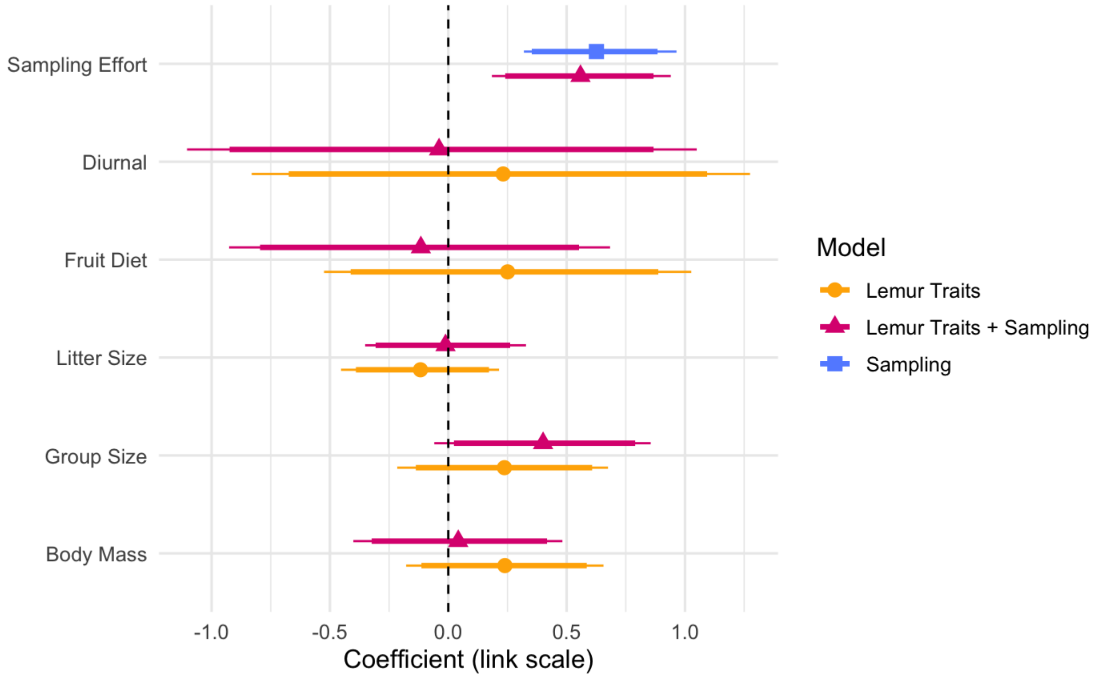

Supplementary Figure 9: Coefficient plot displaying estimates and 95% credible intervals (thin lines) and 90% creedible intervals (thick lines) for the `MCMCglmm` models that account for lemur evolutionary history.

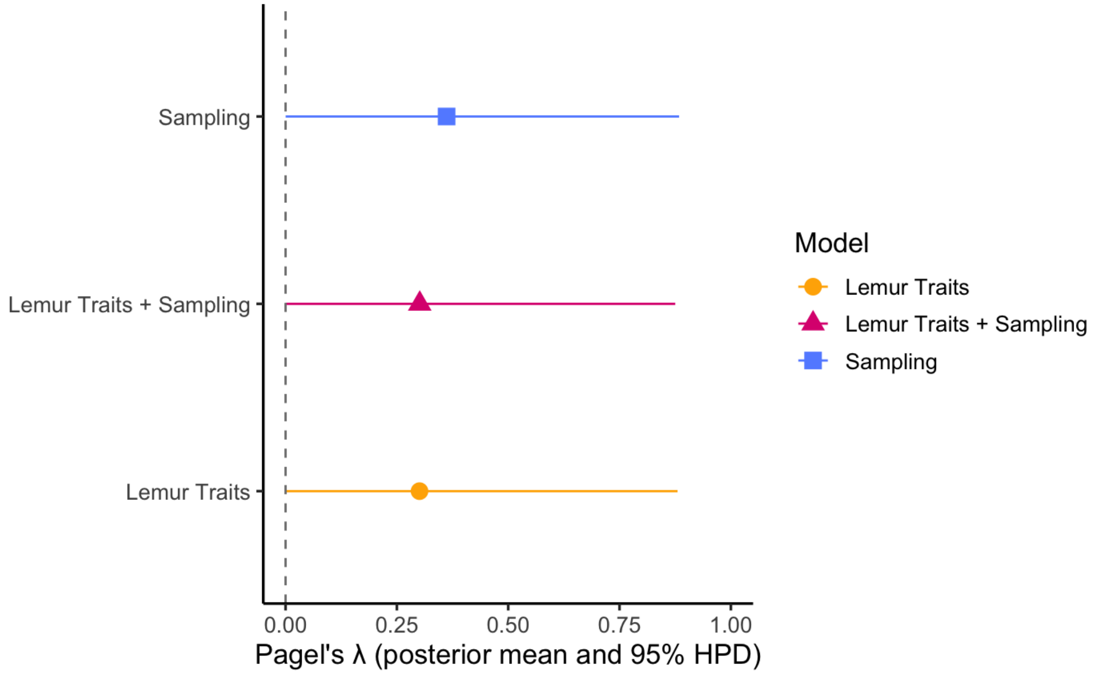

Supplementary Figure 10: Coefficient plot displaying Highest Posterior Density (HPD) intervals for Pagel's λ in the MCMC samples. There is some evidence of phylogenetic signal, but uncertainty is high.

5 Mediation Analyses

5.1 Poisson models

We computed the extent to which sampling effort mediated the influence of binary lemur trait predictors on dietary richness using the mediation package [Tingley et al., 2014, Imai et al., 2010]. Supplementary Tables 15 and 16 display the results of causal mediation analysis using nonparametric bootstrap confidence intervals with the percentile method. We ran 500 simulations for each treatment variable — diurnality and frugivory — on all lemurs, excluding *Indri indri* and *Lemur catta*. Because the R mediation package does not support negative binomial family, we used a Poisson general linear model instead. To confirm that the use of a Poisson model does not qualitatively contradict our other results, we ran the same AIC and pseudo-R-squared bootstrap analyses as described in Section 2.2.3 using the Poisson family. The results do highlight the significance of activity pattern and dietary behavior in predicting lemur dietary richness when sampling was omitted as a predictor (see Supplementary Fig. 2 and Supplementary Tables 2-4). Similarly, when sampling was introduced, the two lemur traits lost their significance. Given this consistency in the conclusions obtained, we feel cautiously justified in employing the Poisson family for mediation analysis.

When activity pattern (diurnal v. nocturnal) was treated as the independent variable, the mediation analysis indicated that a significant portion of its effect on dietary richness was mediated through sampling effort (Table 15). The Average Causal Mediation Effect (ACME) was significant, while the Average Direct Effect (ADE) was negligible, underscoring that the influence of activity pattern was largely indirect and likely reflects oversampling biases towards diurnal species.

In contrast, when diet was analyzed as the treatment variable (Table 16), a different pattern emerged. The ACME remained significant for both control and treated conditions, with estimates of 9.616 and 13.202, respectively, and confidence intervals that excluded zero. This finding confirms that sampling effort also mediated the relationship between frugivory

	Estimate	95% CI Lower	95% CI Upper	p-value
ACME (control)	19.205	4.453	1258.03	0.016 *
ACME (treated)	18.903	5.375	1332.38	0.016 *
ADE (control)	-0.474	-17.704	17.96	0.964
ADE (treated)	-0.777	-62.404	72.38	0.964
Total Effect	18.429	-2.146	1334.62	0.108
Prop. Mediated (control)	1.042	-9.668	4.27	0.100 .
Prop. Mediated (treated)	1.026	-5.430	2.94	0.100 .
ACME (average)	19.054	5.280	1291.87	0.016 *
ADE (average)	-0.625	-40.296	45.25	0.964
Prop. Mediated (average)	1.034	-7.979	3.72	0.100 .

Supplementary Table 15: Mediation results — including Average Causal Mediation Effect (ACME), Average Direct Effect (ADE) and proportion mediated — for sampling effort mediating the relationship between activity pattern and dietary richness (p-value significance codes: 0 '***', 0.001 '**', 0.01 '*', 0.05 '.'). Note that the Poisson general linear model was used; see section 2.2.4 for more details.

and dietary richness. However, unlike diurnality, the total effect of frugivory was significant, suggesting a combined direct and mediated influence on dietary richness. The Average Direct Effect (ADE) was not significant, pointing to variability in the direct pathway.

Notably, the proportion of the effect mediated by sampling effort was substantial, with estimates of 44% for the control condition and 61% for the treated condition, both statistically significant. This indicates that a significant fraction of the apparent relationship between frugivory and dietary richness was explained by sampling biases, mirroring the findings for diurnality, but with a notable direct component that indicates biological relevance.

	Estimate	95% CI Lower	95% CI Upper	p-value
ACME (control)	9.616	3.821	2251.84	0.012 *
ACME (treated)	13.202	5.405	3688.28	0.012 *
ADE (control)	8.524	-12.736	23.87	0.328
ADE (treated)	12.109	-27.315	112.47	0.328
Total Effect	21.725	3.703	3706.62	0.032 *
Prop. Mediated (control)	0.443	0.110	2.10	0.044 *
Prop. Mediated (treated)	0.608	0.191	1.67	0.044 *
ACME (average)	11.409	4.669	2970.06	0.012 *
ADE (average)	10.317	-19.632	67.82	0.328
Prop. Mediated (average)	0.525	0.150	1.86	0.044 *

Supplementary Table 16: Mediation results for diet (frugivory) as the treatment variable predicting dietary richness, as mediated by sampling effort.

5.2 Negative binomial models

Power to detect mediation effects was estimated through Monte Carlo simulation with 1000 draws using the `maczic` package in R. The analysis examined a mediation model where treatment effects on a count outcome operate through two potential variables: diurnal activity patterns and sampling effort. We employed a generalized linear model framework with negative binomial distributions for count outcomes to accommodate overdispersion. The overdispersion parameter theta was set consistent with values estimated in previous negative binomial models of the same outcome. For sampling effort overdispersion the theta of 0.74 as used, calculated the same way as for lemur dietary richness. Sample sizes of 40,000 were specified for 100 dataset simulations. The analysis evaluated both indirect (mediated) effects through each mediator and direct treatment effects, calculating statistical power to detect each pathway.

The power analysis revealed substantial power (92%) to detect the total treatment effect, with adequate power (81%) for detecting direct effects in both control and treatment conditions. However, power to detect mediation effects through the specified pathways was notably low (4% for both conditions), indicating that either the proposed sample size is insufficient to reliably identify indirect effects operating through the mediators, or the priors

Effect Type	Pathway	Estimate	Power
Mediation effect	Control	0.192	0.040
Mediation effect	Treatment	0.099	0.040
Direct effect	Control	0.550	0.810
Direct effect	Treatment	0.457	0.810
Total effect	Combined	0.649	0.920
Mean outcome	Control	20.732	—
Mean outcome	Treatment	21.373	—
Mean non-zero outcome	Control	21.378	—
Mean non-zero outcome	Treatment	22.016	—
Mean percent zero	Control	3.023%	—
Mean percent zero	Treatment	2.920%	—

Supplementary Table 17: Power estimates are based on 100 Monte Carlo simulations with 1,000 draws on 40,000 lemur datasets. Effect estimates represent true mediation and direct effects specified in the simulation. Dashes indicate that power calculations are not applicable for descriptive statistics.

are very non-informative and don't allow accurate interval detetcion. The mediation effect sizes were modest (0.192 in control, 0.099 in treatment) relative to the direct effects (0.550 and 0.457, respectively), suggesting that treatment operates predominantly through direct mechanisms rather than through the examined mediators. Mean outcomes were similar between conditions (20.7 vs 21.4), confirming that zero-inflated modeling approaches may provide minimal advantage over standard count models for these data.

References

Jarrod D. Hadfield. MCMC Methods for Multi-Response Generalized Linear Mixed Models: The MCMCglmm R Package. *Journal of Statistical Software*, 33: 1–22, February 2010. ISSN 1548-7660. doi: 10.18637/jss.v033.i02. URL `https://doi.org/10.18637/jss.v033.i02`.

Kosuke Imai, Luke Keele, and Teppei Yamamoto. Identification, Inference and Sensitivity Analysis for Causal Mediation Effects. *Statistical Science*, 25(1):51–71, February 2010. ISSN 0883-4237, 2168-8745. doi: 10.1214/10-STS321. URL `https://projecteuclid.org/journals/statistical-science/volume-25/issue-1/Identificatio` Publisher: Institute of Mathematical Statistics.

Yi Jin and Hong Qian. U.PhyloMaker: An R package that can generate large phylogenetic trees for plants and animals. *Plant Diversity*, 45(3):347–352, May 2023. ISSN 2468-2659. doi: 10.1016/j.pld.2022.12.007. URL `https://www.sciencedirect.com/science/article/pii/S2468265922001329`.

Dustin Tingley, Teppei Yamamoto, Kentaro Hirose, Luke Keele, and Kosuke Imai. mediation: R Package for Causal Mediation Analysis. *Journal of Statistical Software*, 59:1–38, September 2014. ISSN 1548-7660. doi: 10.18637/jss.v059.i05. URL `https://doi.org/10.18637/jss.v059.i05`.

Nathan S. Upham, Jacob A. Esselstyn, and Walter Jetz. Inferring the mammal tree: Species-level sets of phylogenies for questions in ecology, evolution, and conservation. *PLOS Biology*, 17(12):e3000494, December 2019. ISSN 1545-7885. doi: 10.1371/journal.pbio.3000494. URL `https://journals.plos.org/plosbiology/article?id=10.1371/journal.pbio.3000494`.
